# Coupled endoplasmic reticulum and oxidative stress in pancreatic β cells: mechanisms and therapeutic insights

**DOI:** 10.7717/peerj.21065

**Published:** 2026-04-08

**Authors:** Zhaxicao He, Qian Liu, Xiaohua Yue, Heng Zhao, Jiaorong Yu, Lumei Zhang, Yan Wang, Bing Zhao, Xia Yang, Zhigang Wang

**Affiliations:** 1 School of Clinical Chinese Medicine, Gansu University of Chinese Medicine, Lanzhou, China; 2 TCM Internal Medicine, The Tianshui Hospital of Traditional Chinese Medicine Affiliated to Gansu University of Chinese Medicine, Tianshui, China

**Keywords:** Pancreatic β cells, Endoplasmic reticulum stress, Oxidative stress, Redox homeostasis

## Abstract

Pancreatic β cells maintain glycemic homeostasis through high-rate insulin synthesis and secretion, and their function depends on precise protein folding and the redox microenvironment. In diabetes, inflammation, chronic hyperglycemia, and lipotoxicity disrupt redox homeostasis, with increased reactive oxygen species and compromised antioxidant defenses that directly reduce secretory efficiency and endanger cell survival. Oxidative stress and endoplasmic reticulum stress are tightly coupled. Excessive oxidative load raises folding demand and chronically activates the unfolded protein response, which further perturbs calcium signaling and redox balance to form a vicious cycle. When endoplasmic reticulum stress shifts from adaptive to injurious outputs, β cells undergo a decline in the secretory phenotype, dedifferentiation, and apoptosis, leading to loss of β cell mass and function. As interventions, chemical chaperones and antioxidant strategies can jointly lower ER and oxidative burdens and improve islet function. Tauroursodeoxycholic acid and 4-phenylbutyric acid increase folding capacity and improve metabolic phenotypes across models. Overall, combination approaches centered on folding quality control and redox balance hold translational promise, yet optimal dose and timing, long-term safety, and compatibility with existing glucose-lowering therapies remain to be defined. This review summarizes these mechanistic links and therapeutic advances and discusses key challenges and prospects.

## Introduction

The islet comprises several endocrine cell types, with β cells as the predominant population, alongside α cells, δ cells, and pancreatic polypeptide cells. Their composition and gene features have been delineated by human single cell transcriptomics studies (Lawlor et al., 2017). A small ɛ cell subset is more abundant during embryonic development and secretes ghrelin. In adult islets its proportion is very low yet still detectable (Sakata, Yoshimatsu & Kodama, 2019). As professional secretory cells, β cells markedly increase proinsulin synthesis in response to feeding related stimuli, and in healthy states the production rate reaches approximately six thousand proinsulin molecules per second (Arunagiri et al., 2018). Proinsulin undergoes translocation into the endoplasmic reticulum (ER), signal peptide removal, and formation of three disulfide bonds during folding, which imposes sustained and precise demands on the folding machinery (Liu et al., 2018).

The ER lumen maintains a relatively oxidizing environment that favors disulfide bond formation. Oxidative protein folding generates reactive oxygen species (ROS), creating a close link to ER stress (Malhotra & Kaufman, 2007). Changes in redox homeostasis are sufficient to trigger the unfolded protein response (UPR), and persistent ER stress can induce ROS formation in both the ER and mitochondria, establishing a mutually reinforcing loop (Cao & Kaufman, 2014). Islet cells, particularly β cells, express and activate antioxidant enzymes at comparatively low levels, which explains their high vulnerability to oxidative injury (Lenzen, Drinkgern & Tiedge, 1996). Within physiological limits, ROS also participate in islet signaling and secretion. When present in excess, they damage proteins and provoke β cell dysfunction and apoptosis (Eguchi et al., 2021). Concomitant impairment of proinsulin folding and increased misaggregation indicate that redox imbalance directly perturbs the folding network (Tran et al., 2020).

Therefore, crosstalk between oxidative stress and ER stress is a key determinant of islet homeostasis, with both processes driving each other through folding demand and redox feedback (Cao & Kaufman, 2014; Malhotra & Kaufman, 2007). A moderate UPR expands folding capacity and promotes survival, whereas chronic or excessive activation tends to induce dedifferentiation and apoptosis, thereby advancing the progression of diabetes (Eguchi et al., 2021; Sharma, Landa-Galván & Alonso, 2021). Altogether, attention to the bidirectional regulation between oxidative stress and ER stress, and to their nodal molecules within the islet, provides a solid biological basis for understanding islet homeostatic failure and for designing therapeutic interventions.

## Survey methodology

We undertook a narrative review and searched PubMed and Web of Science from database inception to November 5, 2025, restricting results to English-language publications. Search keywords (and synonyms/MeSH terms where applicable) included: pancreatic islet, beta cell/β-cell, endoplasmic reticulum stress, unfolded protein response/UPR, PERK/EIF2AK3, IRE1α/ERN1, XBP1, ATF6, oxidative stress, reactive oxygen species/ROS, mitochondria/mitochondrial dysfunction, NADPH oxidase/NOX2, calcium homeostasis/SERCA, protein disulfide isomerase/PDI, ER oxidoreductin 1/ERO1, thioredoxin/TXN, peroxiredoxin/PRDX/TXNRD, thioredoxin-interacting protein/TXNIP, proinsulin folding, lipotoxicity, glucotoxicity, ferroptosis, chemical chaperone, antioxidant, and Nrf2.

Audience: This review aims to support islet and β cell researchers and diabetes clinicians.

### Redox regulation as the foundation of islet homeostasis and endocrine performance

#### System level limitations in antioxidant defense and early injury signatures

Pancreatic β cells exist in a metabolically and inflammatory stressed milieu that promotes persistent ROS and RNS accumulation, driven by weak intrinsic antioxidant defenses and amplified by multiple pathways, ultimately impairing hormone secretion and cell viability (Lenzen, Drinkgern & Tiedge, 1996). Compared with other tissues, β cells possess less redundancy for peroxide clearance and repair of oxidative damage, which confers selective vulnerability (Tiedge et al., 1997). Recent changes in oxidative stress markers in pancreatic islets are summarized in Table 1.

Cytokine-driven inflammation initiates islet lipid peroxidation, commonly indexed by malondialdehyde (MDA) and 4-hydroxynonenal (4-HNE) (Rabinovitch et al., 1996). Both markers increase in stimulated human and rodent islets *ex vivo* and correlate with reduced insulin and DNA content. Exogenous addition of either aldehyde reproduces secretory failure and cellular injury in the absence of cytokines, indicating that lipid peroxidation is both a quantitative marker of pathology and an effector that amplifies injury (Suarez-Pinzon, Strynadka & Rabinovitch, 1996). Time-course study shows that rises in MDA and 4-HNE precede overt cell damage, supporting a leading position of lipid peroxidation in the causal chain (Suarez-Pinzon, Strynadka & Rabinovitch, 1996). In metabolic models, β cells from Goto Kakizaki rats display elevated 8-oxoguanine (8-oxoG) and 4-HNE adducted proteins relative to controls, with further increases over age and with islet fibrosis (Ihara et al., 1999). Immunohistochemical detection of 8-oxoG in chronically stressed islets associates with diabetes susceptibility, underscoring nucleic acid oxidation as a key indicator of progression (Ihara et al., 1999). Combined cytokine exposure increases NO and DNA strand breaks, coinciding with worsened secretory dysfunction and cell death and supporting synergistic oxidative–genotoxic stress (Wachlin et al., 2003). DNA breakage activates poly polymerase 1 (PARP1), which depletes nicotinamide adenine dinucleotide and ATP eading to NAD and ATP depletion (Pieper et al., 1999; Suarez-Pinzon et al., 2003). In islet models, loss of PARP1 reduces cytokine induced cell death without necessarily restoring secretion, indicating a partial decoupling between PARP dependent energy collapse and functional impairment (Andreone et al., 2012). In inflammatory and oxidative contexts, protein carbonylation serves as a broad and stable marker of oxidative injury. Proteomics of islets from non-obese diabetic mice shows increased protein carbonylation before clinical onset, providing a sensitive prodromal marker with temporal linkage to subsequent autoimmune destruction (Yang et al., 2022b). 3-nitrotyrosine (3-NT), a specific marker of protein nitration, is increased in cytokine-treated islets and in human type 1 diabetic islets, providing histological evidence of reactive oxygen and nitrogen species–driven protein injury (Martin et al., 2017).

In the low-dose streptozotocin model, islet superoxide dismutase (SOD) progressively declines before overt hyperglycemia, indicating early fragilization driven by reduced antioxidant enzyme capacity (Papaccio et al., 1991). Increasing cytosolic or mitochondrial CAT expression reduces cytokine-induced oxidative toxicity without impairing glucose-stimulated insulin secretion (GSIS), supporting CAT as a key determinant of β-cell resilience (Gurgul et al., 2004; Lortz et al., 2013). At the transcriptional level, Nrf2 activation reduces reactive species and 3-NT, limits oxidative DNA adducts, and mitigates secretion defects, positioning enhanced Nrf2 target expression as a marker of system-level improvement (Yagishita et al., 2014). Pharmacological studies in human islets corroborate this conclusion. Nrf2 activators reduce oxidative stress indices and improve β-cell survival, linking biomarker correction to functional benefit (Masuda et al., 2015). The PRDX/TXN detoxification axis bears much of the peroxide burden; when constrained, ongoing H_2_O_2_ production more readily drives energy failure and cell death, and PRDX/TXN expression or activity may serve as indirect indicators of rising risk (Stancill et al., 2019).

**Figure 1:**
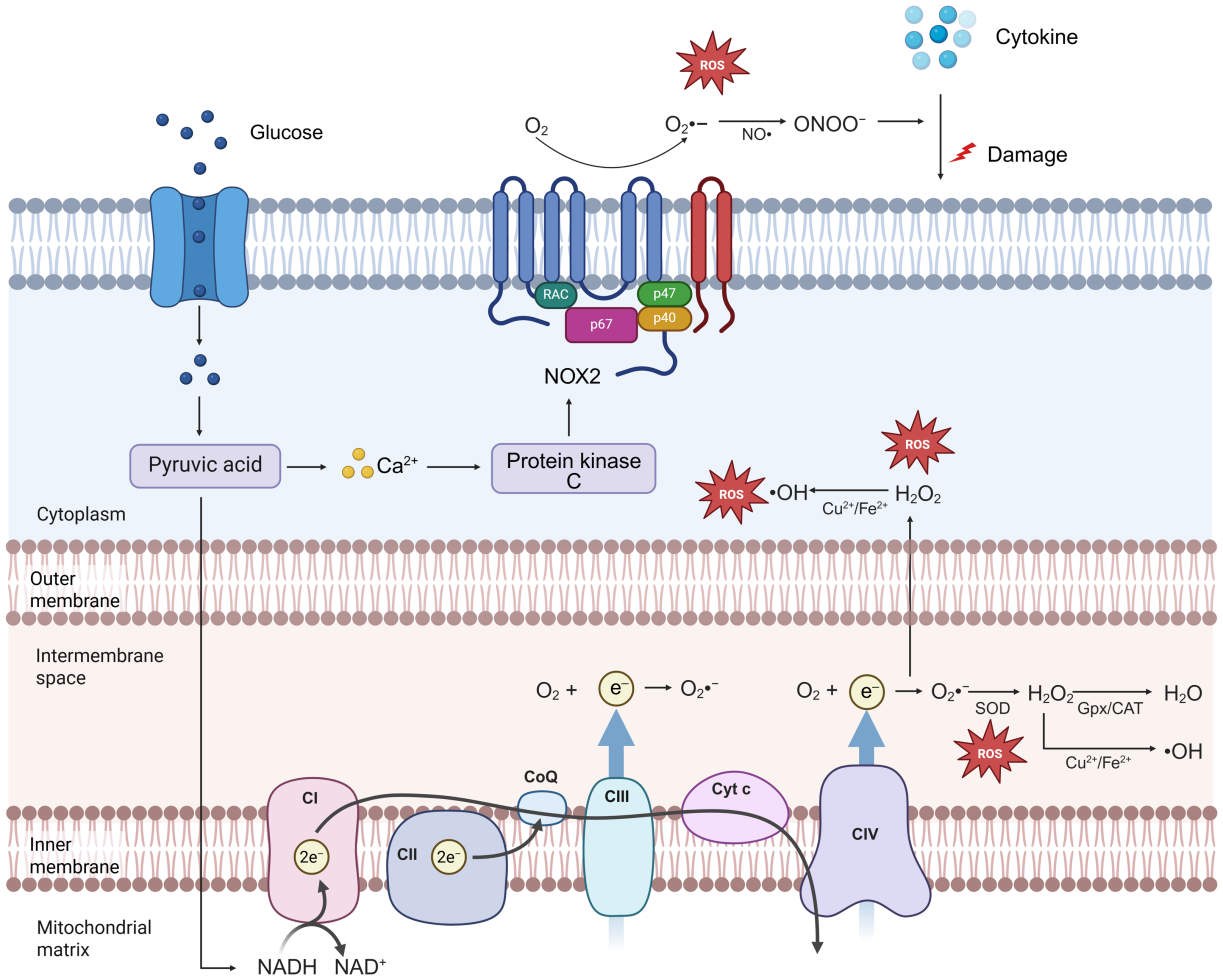
Mitochondrial and NOX sources of ROS in pancreatic β cells. The mitochondrial respiratory chain is a key source of ROS, with electron leakage occurring primarily at complexes I and III (Mukai, Fujimoto & Inagaki, 2022). Although O_2_•− is generated in relatively high amounts, it is extremely unstable; during oxidative phosphorylation, about 4% of consumed oxygen is converted to O_2_•−. O_2_•− is rapidly dismutated by SOD to H_2_O_2_, which is then further reduced to water by GPx or catalase. In the presence of elevated transition metals (*e.g.*, Cu^2^^+^, Fe^2^^+^), H_2_O_2_ can yield the highly oxidative • OH. In β-cells, a rapid increase in glycolytic flux is tightly coupled to mitochondrial oxidative phosphorylation, oxidizing nearly all glucose-derived carbon to CO_2_; consequently, high-glucose metabolism often coincides with mitochondrial ROS production. Meanwhile, expression of SOD and GPx/catalase in β-cells is only ∼30% and ∼5% of that in liver, respectively, resulting in a weaker antioxidant barrier and increased vulnerability to oxidative stress. The glucose-driven rise in intracellular Ca^2^^+^ can activate PKC, which in turn initiates the NOX pathway. Members of the NOX family reside on the plasma membrane or intracellular membranes and generate O_2_•−. β-cells predominantly express NOX2, whose membrane components are gp91phox and p22phox, together with the cytosolic factors p47phox, p67phox, p40phox, and the small GTPases Rac1/2. ONOO^−^, formed by the reaction of O2 • − with NO, is implicated in cytokine-related β-cell injury. GPx, glutathione peroxidase; NOX2, NADPH oxidase 2; Rac1/2, Ras-related C3 botulinum toxin substrate 1/2; ONOO^−^, peroxynitrite.

**Table 1:** Summary of recent alterations in oxidative stress biomarkers in pancreatic islets.

**Biomarker**	**Model (human/animal/cell)**	**Change**	**References**
Malondialdehyde MDA	Human islets with cytokine treatment	Increased levels concurrent with reductions in insulin and DNA content	Rabinovitch et al. (1996)
Malondialdehyde MDA	Rodent islets with combined cytokines	Rises by 4 h and precedes overt cell damage	Suarez-Pinzon, Strynadka & Rabinovitch (1996)
4-Hydroxynonenal 4-HNE	Rodent islets with combined cytokines	Peaks at 8 h and precedes cell damage seen at 16 h	Suarez-Pinzon, Strynadka & Rabinovitch (1996)
4-HNE–modified proteins	Animal model non-obese diabetic rats	Elevated versus controls and increases with disease progression	Ihara et al. (1999)
8-oxo-dG	Animal islets	Positive signal increases and associates with diabetes susceptibility	Ihara et al. (1999)
DNA strand breaks	Cytokine-exposed susceptible islets	Increased strand breaks aligning with impaired secretion and heightened cell death	Wachlin et al. (2003)
PARP activation	Streptozotocin animal islets	Marked activation accompanying DNA damage	Pieper et al. (1999)
NAD levels	Islets under cytokine or free-radical exposure	Decrease linked to excessive PARP activation	Suarez-Pinzon et al. (2003)
ATP levels	Islets with cytokine exposure	Decrease reflecting energetic failure	Andreone et al. (2012)
Protein carbonylation	NOD mouse islets	Elevated before clinical onset and associated with reduced glucose-stimulated insulin secretion	Yang et al. (2022b)
3-Nitrotyrosine 3-NT	Human islets with combined cytokines	Markedly increased and precedes cell damage	Lakey et al. (2001)
3-Nitrotyrosine 3-NT	Human pancreatic tissue type 1 diabetes	Increased staining in islets correlating with disease progression	Martin et al. (2017)

#### Dominant generators of oxidative pressure and roles in stimulus secretion coupling

ROS encompass superoxide (O_2_• −), hydrogen peroxide (H_2_O_2_), and derived oxidants such as hydroxyl radicals (•OH) formed in the presence of redox-active metals (Turrens, 2003). Mitochondria are a dominant source of ROS in β cells, with electron leak at respiratory chain complexes I and III (Fig. 1) (Cadenas & Davies, 2000; Gray & Heart, 2010). Heightened glucose metabolism tightly coupled to oxidative phosphorylation increases mitochondrial ROS, while β cells express only a fraction of the SOD and GPx/CAT levels found in liver, creating a limited antioxidant buffer (Mukai, Fujimoto & Inagaki, 2022; Schuit et al., 1997; Sekine et al., 1994). High-glucose exposure increases mitochondrial ROS in β cells (Bindokas et al., 2003) and impairs insulin secretory function. Pyruvate kinase (PK) shapes ATP/ADP handling by converting phosphoenolpyruvate and ADP to ATP and pyruvate, lowering ADP enough to close ATP-sensitive K^+^ channels before tricarboxylic acid (TCA) flux is fully engaged (Lewandowski et al., 2020; Merrins et al., 2022). This highlights a spatiotemporal compartmentalization of glucose metabolism and stimulus–secretion coupling that intersects with ROS generation (Ho et al., 2023).

Glucose-evoked Ca^2^^+^ entry activates PKC to stimulate NOX; β cells express NOX2 (gp91phox/p22phox with p47phox, p67phox, p40phox, and Rac1/Rac2), which generates O_2_• − from plasma and intracellular membranes, and NOX inhibition (pharmacologic or genetic) perturbs Ca^2^^+^ dynamics and dampens GSIS (Morgan et al., 2007; Morgan et al., 2009; Newsholme et al., 2009). In β cells, NOX-derived ROS can act as metabolic signals during GSIS (Leloup et al., 2009), yet animal data also suggest a restraining and pathogenic role: islets from Nox2-null mice show that NOX2-derived O_2_• − limits GSIS and contributes to cytokine-induced secretory defects, and NOX2 components are upregulated in Zucker diabetic fatty rats *versus* lean controls (Li et al., 2012; Syed et al., 2011; Xiang et al., 2010). However, De Souza et al. (2017) report that NOX2 is not responsible for glucose-induced oxidative stress or β-cell dysfunction. Taken together, NOX’s precise role in β-cell metabolism remains unsettled.

Peroxynitrite (ONOO^−^) forms when O_2_• − reacts with NO and has been linked to cytokine-associated β-cell injury (Lakey et al., 2001). Yet cytokines alone do not produce detectable ONOO^−^ in β cells, and forced ONOO^−^ generation *via* exogenous O_2_• − plus NO can scavenge NO and attenuate NO-driven damage, supporting NO as the principal mediator of cytokine toxicity while ONOO^−^ effects are context-dependent and potentially protective (Broniowska, Mathews & Corbett, 2013). These findings position NO as the principal mediator of cytokine toxicity, with ONOO^−^ formation exerting context-dependent, potentially protective effects.

The PRDX/TXN system comprises multiple PRDX isoforms that detoxify H_2_O_2_, with oxidized PRDX recycled by TXN and TXNRD using NADPH, forming a frontline H_2_O_2_ sink across compartments (Stancill & Corbett, 2021). In β cells, cytosolic PRDX1/2, mitochondrial PRDX3, and ER-localized PRDX4 are induced by inflammatory or oxidative stressors including cytokines and diabetogenic toxins, and overexpression of these PRDXs mitigates diverse β-cell injuries (Bast et al., 2002; Mehmeti et al., 2014; Stancill et al., 2020). Likewise, TXN overexpression blunts hyperglycemia progression in db/db mice (Yamamoto et al., 2008), and cytosolic thioredoxin-1 (Trx1) can be secreted from β cells during hypoxia or glucose stimulation, with exogenous Trx1 improving hypoxia induced secretory defects, suggesting a paracrine contribution to β cell function (Hanschmann et al., 2020). Thioredoxin interacting protein (TXNIP; also TBP-2/VDUP1) is robustly induced by glucose in human islets at the transcriptional level and, by binding TXN, suppresses TXN activity, shifts the cellular redox poise, and provokes oxidative stress; TXNIP is elevated in diabetic β cells irrespective of obesity, whereas TXNIP loss is cytoprotective (Cha-Molstad et al., 2009; Shalev, 2014). Importantly, β cells can rely on the PRDX/TXN system to remove micromolar H_2_O_2_, providing compensatory defense when classical antioxidant enzymes are scarce (Thielen & Shalev, 2018).

### Endoplasmic reticulum stress within the professional secretory milieu of the beta cell

Pancreatic β cells carry a heavy burden of insulin synthesis and secretion. Their ER folding environment relies on precise coordination between redox homeostasis and calcium homeostasis. Chronic imbalance triggers ER stress and engages the three canonical pathways of the UPR, which together determine adaptation or apoptosis (Fonseca, Gromada & Urano, 2011). Multiple experimental studies show that sustained ER stress and oxidative stress amplify one another and culminate in β cell dysfunction and loss, a central pathological step in the onset and progression of diabetes (Song et al., 2008).

#### Hallmarks of heightened folding demand and proteostasis imbalance across metabolic and inflammatory contexts

Under metabolic overload and toxic stimuli, β cells first display increased demand for chaperones within the ER lumen together with accumulation of misfolded proteins. Typical markers include upregulation of binding immunoglobulin protein (BiP), phosphorylation of eukaryotic initiation factor 2 alpha (eIF2α), splicing of X box binding protein 1 (XBP1), and induction of CCAAT enhancer binding protein homologous protein (CHOP). Prolonged exposure to saturated fatty acids elicits these changes and provokes apoptosis in islet cells, indicating that lipotoxicity directly drives ER stress elevation (Boslem et al., 2011). High glucose and ROS enhance CHOP and Jun N terminal kinase (JNK) responses, reduce secretory function, and intensify cell death. These findings indicate that glucotoxicity raises ER stress through combined oxidative injury and folding load (Lin et al., 2012). Human islet amyloid polypeptide readily forms misfolded aggregates within β cells and directly triggers ER stress, thereby weakening insulin biosynthesis and secretion (Huang et al., 2007).

Inflammatory signaling markedly amplifies ER stress through NO and ER calcium depletion. Cytokine induced NO downregulates sarco ER calcium ATPase 2b (SERCA2b) and depletes ER calcium stores, which in turn activates protein kinase RNA like ER kinase (PERK) and inositol requiring enzyme 1 alpha (IRE1α) pathways and induces CHOP. This chain of events has been validated in rodent and human islet cells, demonstrating direct transduction of inflammatory stress into ER stress signaling (Brozzi et al., 2015). Earlier experiments established that low dose NO can trigger CHOP through ER calcium depletion and lead to β cell apoptosis, underscoring the central role of ER stress in β cell injury (Oyadomari et al., 2001). In disease models, ER stress markers are already prominent in β cells before clinical onset in non-obese diabetic mice, indicating that ER stress is an early event in the disease course (Tersey et al., 2012). Elevated ER stress markers have also been documented pathologically in human type 1 diabetes (T1D) islets (Marhfour et al., 2012).

Genetic and pharmacological evidence converge to show that persistent ER stress is tightly linked to β cell decompensation. The eIF2α branch, which limits translational load, is essential for β cell survival. Its impairment worsens glucose tolerance and aggravates ER dysregulation (Scheuner et al., 2005). Deletion of CHOP reduces oxidative injury and improves β cell structure and function, identifying the proapoptotic arm of the UPR as a key driver of islet failure (Song et al., 2008). Integrity of IRE1α and XBP1 is indispensable for preserving secretory capacity and restraining oxidative stress. β cell specific weakening of this pathway causes secretory defects and an oxidative inflammatory phenotype, while antioxidant intervention partially corrects dysfunction (Hassler et al., 2015). IRE1α and XBP1 also induce members of the protein disulfide isomerase family (PDI) to maintain oxidative folding of proinsulin. Loss of this regulation directly lowers insulin output and exacerbates ER stress (Tsuchiya et al., 2018). Chemical chaperones that restore UPR balance markedly reduce the incidence of autoimmune diabetes, providing system level support for a pathogenic role of ER stress in the disease course (Engin et al., 2013).

#### Oxidative load reinforcing ER stress and remodeling of quality control networks

**Figure 2:**
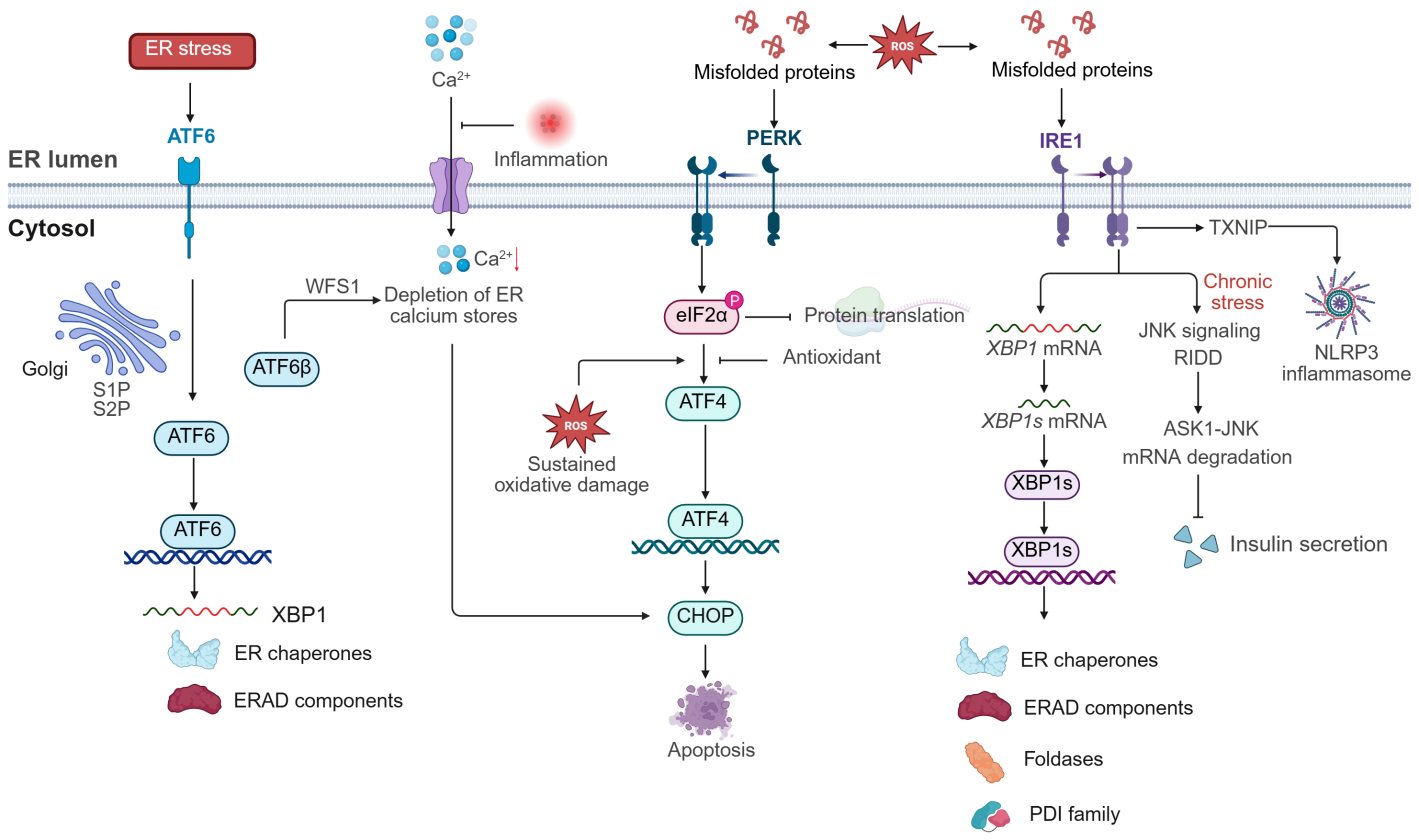
Oxidative stress driven UPR dynamics in β cells. Oxidative stress–induced misfolding promotes PERK oligomerization and kinase activation, leading to eIF2α phosphorylation and rapid attenuation of global translation. When damage persists, sustained ATF4 and CHOP elevation drives a switch of the UPR from adaptive to pro-apoptotic, manifested by loss of islet secretory phenotype and reduced cell mass. Antioxidant interventions partially improve glucose tolerance and cell survival. Inflammation-induced NO suppresses SERCA and depletes ER Ca^2^^+^, rapidly inducing CHOP and triggering ER stress–related apoptosis. Accumulation of misfolded proteins together with increased oxidative stress promotes high-order oligomerization of IRE1α at the ER membrane, activating its kinase and RNase. With brief activation, IRE1α splices XBP1 to generate XBP1s, which upregulates chaperones, foldases, and ERAD components. XBP1s also enhances expression of multiple protein disulfide isomerases, including PDI family members, improving proinsulin oxidative folding and stabilizing secretory throughput. As stress persists and oxidative burden accumulates, IRE1α RNase output shifts from adaptive XBP1s toward RIDD and JNK-linked injury programs, characterized by degradation of ER-targeted mRNAs and activation of ASK1–JNK and inflammatory signaling; in β-cells this leads to reduced insulin mRNA and declining secretion. IRE1α further elevates TXNIP and activates the NLRP3 inflammasome. ATF6α exists in detectable active forms in β-cells and increases with ER stress, inducing GRP78 and key ERAD components to boost folding and clearance capacity. ATF6 and XBP1 exhibit functional crosstalk; together they more efficiently drive chaperone and quality-control networks, helping maintain secretory homeostasis under oxidative stress. ATF6β supports survival by inducing genes such as WFS1, which helps preserve ER Ca^2^^+^ stores and stress recovery. SERCA, sarcoplasmic endoplasmic reticulum Ca^2^^+^ ATPase; RIDD, regulated IRE1-dependent decay; ASK1, apoptosis signal-regulating kinase 1; TXNIP, thioredoxin-interacting protein; NLRP3, NLR family pyrin domain containing 3; GRP78, glucose-regulated protein 78; PDI, protein disulfide isomerase; WFS1, wolframin.

Coupling between oxidative stress and oxidative folding in the ER is a central driver of stress escalation in β cells (Fig. 2). Disulfide bond formation within the ER lumen depends on an oxidizing environment and on the PDI network. Redox imbalance increases misfolding and consumes chaperone capacity, which amplifies input into the UPR. In β cells, the IRE1α and XBP1 axis is activated by glucose and secretory load to expand folding capacity while constraining oxidative stress driven decompensation. Attenuating this axis upregulates oxidative and inflammatory gene programs and impairs glucose tolerance, indicating that in physiology it serves both pro folding and antioxidative roles (Hassler et al., 2015). Further experiments show that IRE1α and XBP1 directly increase expression of multiple PDI family members and maintain efficient oxidative folding of proinsulin, which provides a molecular basis for the interplay between oxidative pressure and ER stress (Tsuchiya et al., 2018).

Oxidative pressure promotes misfolding and calcium efflux from the ER, which fosters oligomerization of PERK and activates its kinase activity. PERK then phosphorylates eIF2α to rapidly lower global translation, creating time for repair of the folding machinery. This early inhibitory adaptation is especially important in β cells (Scheuner et al., 2005; Zhang et al., 2006). When oxidative damage persists, activating transcription factor 4 (ATF4) and CHOP remains elevated downstream of the eIF2α pathway, shifting the UPR from adaptation to a proapoptotic program that presents as loss of secretory phenotype and cell number (Back et al., 2009). Antioxidant intervention partly corrects glucose intolerance and cell death in mice that lack eIF2α phosphorylation, which identifies oxidative pressure as a key upstream driver of the transition of the PERK/eIF2α/ATF4/CHOP cascade from protection to injury (Back et al., 2009). Inflammatory NO suppresses SERCA2b, depletes ER calcium stores, rapidly induces CHOP, and triggers ER stress related apoptosis. These findings demonstrate efficient conversion of inflammatory oxidative cues into a death program through the PERK/eIF2α/ATF4/CHOP cascade (Oyadomari et al., 2001). In lipotoxic models, inhibition of mitochondrial ROS reduces upregulation of ER chaperones and CHOP and lowers apoptosis in INS1 cells, which places excess ROS as a direct upstream driver of the PERK and CHOP arm (Lin et al., 2012). Changes in energy and nutrient status also regulate translation and ER load through the PERK/eIF2α branch, indicating that metabolic and oxidative stresses converge on this pathway (Gomez et al., 2008).

Accumulation of misfolded proteins and increased oxidative pressure promote formation of high order IRE1α oligomers at the ER membrane and activate its kinase and RNase activities. During brief activation, IRE1α splices XBP1 to generate the spliced form XBP1s, which induces chaperones, folding enzymes, and ER associated degradation (ERAD) components. This expands folding and clearance capacity in β cells and limits oxidative injury, as demonstrated in β cell specific genetic models and in human islets (Hassler et al., 2015). XBP1s also enhances expression of several PDI family members, which improves oxidative folding of proinsulin and stabilizes secretory throughput. These observations establish a direct connection between the IRE1α and XBP1 axis and ER oxidative folding at the level of folding enzymes and disulfide bonds (Tsuchiya et al., 2018). With persistent stress and rising oxidative load, the RNase output of IRE1α shifts from XBP1s toward regulated IRE1 dependent decay (RIDD) and toward JNK linked injury. This includes degradation of a set of ER directed mRNAs and activation of apoptosis signal regulating kinase 1 (ASK1), JNK, and inflammatory pathways, which lowers insulin mRNA and depresses secretion. Chronic hyperglycemia and forced IRE1α expression both reveal this transition from adaptation to injury (Lipson, Ghosh & Urano, 2008). IRE1α also induces TXNIP and activates the NLRP3 inflammasome, which links ER stress to oxidative inflammation and proapoptotic signaling. Inhibiting TXNIP or the inflammasome markedly reduces β cell death and identifies a crossroad between oxidative signals and the IRE1 pathway (Oslowski et al., 2012). In mice with β cell specific deletion of Ire1α, glucose stimulated insulin secretion and biosynthesis decline while oxidative stress markers rise. Antioxidant treatment partially restores glucose tolerance and secretion, which again highlights the protective role of this axis in limiting oxidation driven ER stress (Hassler et al., 2015).

Activating transcription factor 6 alpha (ATF6α) is present in an active form in β cells and increases with heightened ER stress. ATF6α upregulates glucose regulated protein 78 and key ERAD components to raise folding and clearance capacity, which supports handling of misfolding caused by oxidation (Teodoro et al., 2012). ATF6 and XBP1 display functional crosstalk. Together they more efficiently drive chaperones and quality control networks and help sustain secretory homeostasis under oxidative pressure. This cooperation has been systematically elucidated in islet cell models (Sharma, Darko & Alonso, 2020). Activating transcription factor 6 beta (ATF6β) contributes to survival by inducing genes such as Wolfram syndrome 1 (WFS1). WFS1 supports ER calcium storage and recovery from stress and provides additional homeostatic insurance in chronic oxidative and folding challenges (Odisho, Zhang & Volchuk, 2015). In autoimmune diabetes models, chemical chaperones that rebalance the UPR reduce diabetes incidence and improve islet architecture and secretion. This protection depends on the ATF6 axis and suggests that strengthening ATF6 and its cooperating network can shift the UPR from terminal injury back to the adaptive range in settings driven by oxidation and inflammation (Engin et al., 2013).

In summary, excessive oxidative folding mediated by ER oxidoreductin 1 (ERO1) and PDI can cause disulfide mismatching and substrate congestion, while inflammatory oxidants inhibit SERCA and deplete ER calcium. Together these processes reduce the availability of BiP and expose luminal regulatory domains of PERK, IRE1α, and ATF6α, which activates these sensors. Early in stress, PERK lowers translation through eIF2α, IRE1α expands folding capacity through XBP1s, and ATF6α upregulates chaperones and ERAD to remodel quality control. These branches cooperate to reduce excess oxidative byproducts and maintain secretory homeostasis. When oxidative load persists and exceeds compensatory thresholds, the PERK/eIF2α/ATF4/CHOP arm becomes proapoptotic, IRE1α favors RIDD and JNK signaling, and the survival buffer provided by ATF6β is breached, culminating in loss of β cell function and mass.

### Converging cellular stress programs and the progression from functional adaptation to failure

#### Adaptive responses that preserve identity and secretory capacity and define a reversible window

**Figure 3:**
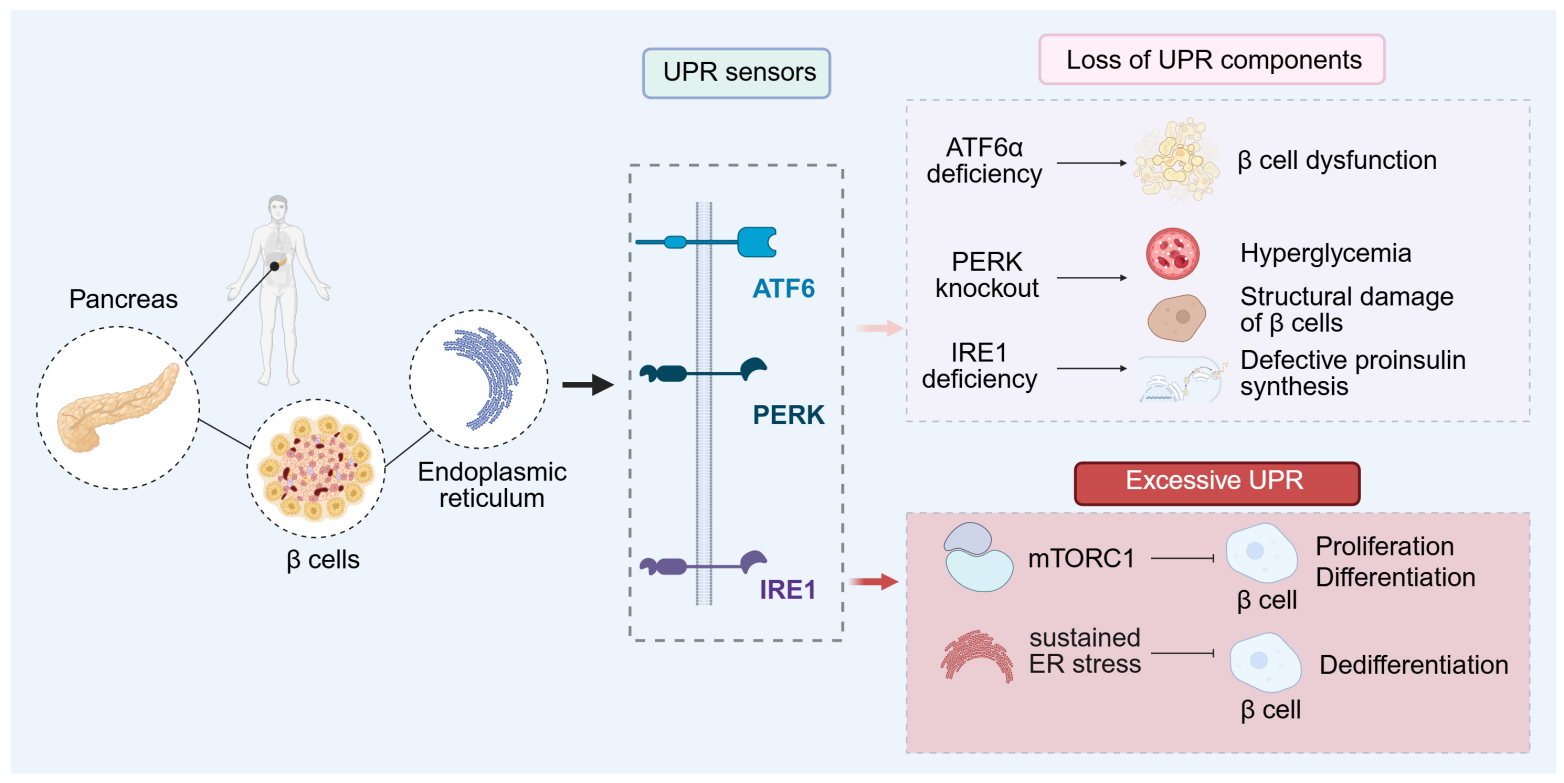
ER homeostasis and UPR regulation in beta cell proteostasis. Maintaining ER homeostasis is essential for the fidelity of protein folding, assembly, and secretion. β-cells engage the UPR to ensure proper folding of proinsulin within the ER. PERK knockout causes hyperglycemia accompanied by disruption of islet and β-cell architecture; PERK mutations lead to β-cell failure. ATF6α deficiency results in β-cell dysfunction. Loss of IRE1 produces defective proinsulin biosynthesis and diabetes. Excessive UPR suppresses mTORC1, thereby limiting β-cell proliferation and differentiation; sustained ER stress can also induce partial dedifferentiation of adult β-cells. ATF6α, activating transcription factor 6 alpha; IRE1, inositol requiring enzyme 1; PERK, protein kinase R like ER kinase; mTORC1, mechanistic target of rapamycin complex 1.

The ER is the principal site where nascent polypeptides such as proinsulin are folded and assembled, acquiring their correct tertiary structure for efficient secretion. Consequently, ER homeostasis is critical for the fidelity of protein folding, assembly, and export (Wang & Kaufman, 2012). Secretory cells in general depend on a well-balanced ER milieu, but pancreatic β cells are especially susceptible to ER stress because proinsulin synthesis and turnover are exceptionally high. By integrating blood glucose with neural and hormonal cues, β cells adjust insulin biosynthesis and release to maintain systemic glucose balance. In humans after feeding, surging secretory demand accelerates insulin production, imposing considerable stress on the ER folding machinery (Chen et al., 2022). To cope, β cells activate the UPR to secure proper proinsulin folding within the ER (Fig. 3). This regulated program is essential for β cell viability and function (Mbara et al., 2024). Gain-/loss-of-function evidence in animals and humans shows that adaptive UPR transducers are decisive determinants of β cell performance (Lee et al., 2022). For instance, deleting PERK in mice causes hyperglycemia from youth into adulthood and disrupts islet/β-cell architecture (Gao et al., 2012), whereas PERK mutations in humans lead to β cell failure and neonatal diabetes (Delépine et al., 2000), highlighting its role in islet maintenance. The second sensor, activating transcription factor 6 (ATF6), is likewise required: ATF6α-null mice develop impaired glucose tolerance on a high-fat diet due to β cell insufficiency (Usui et al., 2012); ATF6 genetic variants associate with prediabetes in Han Chinese (Gu et al., 2014) and with type 2 diabetes in Pima Indians (Thameem et al., 2006). The third branch, IRE1α, is necessary for GSIS and β cell protection; IRE1 deficiency precipitates diabetes owing to defective proinsulin synthesis (Hassler et al., 2015).

Akita mice harbor the Ins2 Cys96Tyr substitution, which disrupts disulfide bond formation, yields misfolded insulin, and provokes ER stress. In Akita and in Lepr db/db mice on the C57BLKS background, the ER chaperone BiP is already elevated before hyperglycemia appears. BiP levels track with β cell proliferation, and pharmacologic relief of ER stress with a chemical chaperone suppresses this proliferative response (Sharma et al., 2015). These observations imply that, under rising insulin demand, UPR signaling can deliver pro-proliferative cues to expand β cell mass. *In vitro*, human β cells also exhibit limited proliferation under ER stress (Sharma et al., 2015), though whether this translates *in vivo* is uncertain. Unlike mice, adult human β cells rarely divide (Mezza & Kulkarni, 2014; Perl et al., 2010). In xenograft models where human islets are transplanted into immunodeficient mice, either a high-fat diet or an insulin-receptor antagonist increases proliferation of the host mouse β cells but leaves human grafts unaffected (Dai et al., 2016).

In neonatal-oriented models, excessive UPR activity in Akita mice suppresses mTORC1, thereby constraining β cell proliferation and population expansion (Riahi et al., 2018). Induced pluripotent stem cells from patients with dominant insulin mutations can be differentiated into β cells and transplanted into immunodeficient mice; such grafts display heightened ER stress with reduced β cell function and number (Balboa et al., 2018; Zhang et al., 2024). The numerical decline has been attributed either to lower proliferation *in vitro* accompanied by weakened mTORC1 signaling *in vivo* (Balboa et al., 2018), or to dedifferentiation marked by ALDH1A3-positive, NKX6.1-positive cells (Zhang et al., 2024). Neither study detected a change in apoptosis. Likewise, β cell–specific deletion of IER3IP1 during mouse embryogenesis (Yang et al., 2022a) or deletion of Perk (Zhang et al., 2006) diminishes β cell mass due to impaired proliferation or differentiation. Overall, persistent and severe ER stress during fetal and early postnatal life chiefly limits β cell expansion by suppressing proliferation and differentiation, with apoptosis contributing to a lesser extent. By contrast, in adults, ER stress driven by a high biosynthetic load terminates β cell proliferation and may help preserve identity (Szabat et al., 2016). Conversely, conditional Ins2 deletion on an Ins1-null background acutely lowers insulin output and downregulates the UPR prior to hyperglycemia, transiently boosting β cell proliferation (Szabat et al., 2016). Episodes of excessive ER stress can also elicit partial dedifferentiation in adult β cells. Time-course studies using cyclosporin A in mouse and rat β cells show coordinated downregulation of identity/function genes (Ins1, Ins2, NKX2.2, PDX1, MAFA, and SLC2A2/GLUT2), with expression restored upon stressor removal, indicating plasticity (Chen et al., 2022; Pirot et al., 2007). *Ex vivo* human islets align with this view: in insulin resistance or impaired glucose tolerance, dedifferentiated β cells, insulin/glucagon double-positive cells, and insulin/keratin-19 double-positive cells are observed but are not evident in individuals with normal glucose tolerance (Mezza et al., 2019). Because most human data are cross-sectional and rely on surgical or donor material, the reversibility of de- or trans-differentiation remains difficult to establish; transplantation of primary or stem cell–derived human islets into mice may help resolve this.

In summary, an intact adaptive UPR is a prerequisite for normal β cell function. Early, reversible ER-stress–induced dedifferentiation may define a therapeutic window in polygenic diabetes, during which lowering ER load could permit recovery. In non-obese diabetic mice, β cell–specific deletion of Ire1 after birth first triggers dedifferentiation and hyperglycemia, followed by a return to normoglycemia and protection from T1D, consistent with immune evasion, reduced insulitis, and limited apoptosis. A separate study shows that deleting Atf6 before insulitis initiates early β cell senescence, recruits M2 macrophages to clear terminally senescent cells, enhances immune surveillance, and protects remaining non-senescent β cells (Lee et al., 2023a). Notably, β cell–specific deletion of Ern1 or Atf6 is protective in NOD mice (Lee et al., 2020; Lee et al., 2023a) yet can precipitate diabetes on a C57BL/6 background (Tsuchiya et al., 2018), underscoring the strong context and genetic dependence of UPR outcomes.

#### Maladaptive remodeling leading to dedifferentiation apoptosis and sustained functional loss

*In vitro*, repeated supraphysiological ER stress progressively diminishes the ability of β cells to regain function (Chen et al., 2022). Cells may then mount a maladaptive UPR culminating in apoptosis, or enter irreversible terminal dedifferentiation accompanied by profound functional decline (Bensellam, Jonas & Laybutt, 2018).

Although T1D and type 2 diabetes (T2D) are both polygenic, their etiologies differ. Numerous studies report heightened ER stress in β cells in both conditions, compromising insulin secretion (Shrestha et al., 2021). In T1D, pro-inflammatory cytokines such as IL-1β and IFN-γ induce ER stress in β cells (Cardozo et al., 2005), activating ER membrane-sensor pathways and weakening chaperone defenses (Cardozo et al., 2005). Consistently, patient islet sections show elevated ATF3, CHOP, and BiP (Hartman et al., 2004; Marhfour et al., 2012).

T2D, characterized by insulin resistance and metabolic stress in pancreatic and peripheral tissues, imposes persistent workload on β cells, provoking sustained ER stress and eventual failure. Declining β cell mass is linked primarily to increased apoptosis (Butler et al., 2003; Donath & Halban, 2004), with ER stress as a key precipitant (Shrestha et al., 2021). In line with this, CHOP is markedly elevated in islets from individuals with T2D: compared with obese non-diabetic subjects, obese diabetic individuals display ∼sixfold higher perinuclear CHOP (Huang et al., 2007). Deleting Ddit3 (encoding CHOP) protects β cells from apoptosis and improves hyperglycemia across several mouse models, including Lepr db/db mice, high-fat diet paradigms, and Akita mice (Oyadomari et al., 2002; Song et al., 2008). Remarkably, β cell–specific conditional CHOP deletion prevents hepatic steatosis induced by high-fat feeding or aging and lowers insulin transcripts by ∼75%, yet does not alter glucose tolerance or body weight (Yong et al., 2021). Forced expression of ATF4 suppresses β cell identity genes and impairs oral glucose tolerance, at least partly *via* reduced responsiveness to GLP-1 and GIP; this incretin desensitization is mediated by upregulated PDE4D and attenuated cAMP signaling (Lee et al., 2023b). Consistently, ER stress is evident in Lepr db/db islets, with XBP1, DNAJC3, ATF4, CHOP, and BiP upregulated; corresponding increases in DNAJC3, CHOP, and BiP are seen in pancreatic sections from individuals with T2D (Laybutt et al., 2007). Recent work shows that from normal glucose tolerance to impaired glucose tolerance and then to T2D, abnormalities in proinsulin-to-insulin indices and ER stress markers intensify progressively (Huang et al., 2007) and correlate with early loss of β cell identity (Brusco et al., 2023). Overall, T2D progression features rising expression of ER-stress–related genes together with increased β cell workload due to high insulin demand and insulin resistance. This trajectory culminates in identity and functional loss, supporting ER stress as a central driver of β cell apoptosis in polygenic diabetes.

Accordingly, reducing ER stress should lessen β cell burden and delay failure in T2D. Augmenting UPR capacity is therefore a plausible preventive strategy for polygenic diabetes. Reported approaches include small molecules with chaperone-like activity, tauroursodeoxycholic acid (TUDCA) and 4-phenylbutyric acid (PBA), to boost ER folding capacity (Welch & Brown, 1996; Yadav et al., 2019). In humans, PBA partially mitigates lipid-induced insulin resistance and β cell dysfunction. In leptin-deficient ob/ob mice, both PBA and TUDCA reduce ER stress, improve glycemic control, and enhance systemic insulin sensitivity (Ozcan et al., 2006), suggesting that reinforcing ER adaptability with chemical chaperones carries meaningful antidiabetic potential.

### Proinflammatory cytokine–induced ER and oxidative stress in pancreatic β cells

In pancreatic islets, proinflammatory cytokines such as IL-1β, TNF-α and IFN-γ drive β-cell dysfunction and death by inducing both endoplasmic reticulum ER stress and oxidative stress (Eizirik, Colli & Ortis, 2009). Among these, IL-1β has been identified as a key initiator linking the inflammatory microenvironment to ER stress in β cells. In primary rat β cells and INS-1E cells, combined exposure to IL-1β and IFN-γ markedly upregulates iNOS and increases NO production, which *via* an NO-dependent mechanism decreases mRNA and protein levels of the ER Ca^2+^ pump SERCA2b, depletes ER Ca^2+^ stores, activates ER stress pathways including IRE1α, PERK and CHOP, and ultimately induces apoptosis and necrosis (Cardozo et al., 2005). IL-1β alone is sufficient to induce ER Ca^2+^ efflux and JNK activation in rat primary β cells and MIN6 cells, accompanied by PERK phosphorylation, and pharmacological JNK inhibition significantly attenuates IL-1β-induced apoptosis (Wang et al., 2009). In mouse β-cell models treated with NO donors or engineered to overexpress iNOS, NO induces BiP and CHOP expression and activates caspase-12, whereas genetic or pharmacological inhibition of the PERK–CHOP axis partially prevents NO-induced apoptosis, supporting NO-mediated ER stress as a critical intermediary of cytokine toxicity (Oyadomari et al., 2001). In INS-1E cells, apoptosis triggered by an IL-1β-dominated cytokine cocktail depends strongly on NF-κB-driven iNOS induction and NO production and is accompanied by activation of canonical ER stress markers (Kharroubi et al., 2004). In human islets and β-cell lines, IL-1β in combination with other proinflammatory cytokines rapidly activates 12-lipoxygenase, leading to generation of 12-HETE, which in the absence of additional inflammatory stimuli is sufficient to upregulate NOX1 and induce ROS production; inhibition of 12-lipoxygenase or NOX1 markedly reduces ROS burden and lowers caspase-3 cleavage and β-cell death, indicating that the IL-1β-dominated inflammatory milieu amplifies oxidative stress through a 12-lipoxygenase–NOX1–ROS axis that cooperates with NO-induced ER Ca^2+^ dysregulation to establish a positive feedback loop (Chen et al., 2005; Weaver et al., 2012).

TNF-α itself exerts relatively weak acute direct toxicity on β cells, but in combination with IL-1β and IFN-γ it markedly amplifies the above stress burden. In human and rodent islets, this cytokine mixture enhances IL-1β-driven transcriptional responses through TNFR1-mediated NF-κB and JNK signaling, leading to upregulation of iNOS and multiple inflammatory mediators and cooperative impairment of SERCA2 function, thereby exacerbating ER Ca^2+^ dysregulation and UPR load (Cardozo et al., 2005; Eizirik, Colli & Ortis, 2009). In mouse islets and MIN6 cells, triple stimulation with IL-1β, TNF-α and IFN-γ induces PERK and eIF2α phosphorylation, JNK activation and Serca2b downregulation. When iNOS is deleted or NO synthesis is inhibited, ER stress still occurs, but the UPR is partially shifted from a CHOP- and ATF3-dominated proapoptotic branch toward an adaptive branch characterized by induction of molecular chaperones and folding enzymes, suggesting that NO primarily influences UPR branch selection rather than its initiation (Chan et al., 2011). In rat and human islets and EndoC-βH1 cells, the same triple cytokine stimulus also reveals species differences. In rat islets, ER stress is highly dependent on NO and SERCA2 inhibition, whereas in mouse and human β cells JNK and CHOP play more central roles, and JNK inhibition or CHOP knockdown substantially reduces apoptosis, indicating that this cytokine combination establishes a lethal UPR program centered on the JNK–CHOP axis in these species (Brozzi et al., 2015).

With respect to oxidative stress, combined stimulation with IL-1β, TNF-α and IFN-γ in INS-1 cells and human islets upregulates NOX1 and promotes ROS production in a 12-lipoxygenase–dependent manner, driving expression of inflammatory mediators such as MCP-1 and activation of caspase-3 (Weaver et al., 2012). NOX inhibitors or 12-lipoxygenase inhibitors markedly lower ROS levels and protect β cells, indicating that this composite proinflammatory signal is converted into NADPH oxidase-dependent oxidative stress that synergistically amplifies toxicity at both the ER and mitochondrial levels, with IFN-γ playing a key role in enhancing transcriptional responses *via* STAT1-related pathways and sustaining the overall stress burden (Brozzi et al., 2015; Eizirik, Colli & Ortis, 2009; Weaver et al., 2012).

### Translational strategies to restore islet resilience through folding quality control and redox balance

During the progression of diabetes, pancreatic β cells bear dual burdens from oxidative stress and ER stress (Oslowski & Urano, 2011). These stresses amplify each other and drive β cell functional decline, dedifferentiation, and death, ultimately reducing islet functional reserve (Karaskov et al., 2006). Experimental and clinical evidence indicates that certain drugs and natural compounds with antioxidant or chemical chaperone properties can lower ROS, improve the folding environment, and remodel UPR pathways. Through these actions they directly or indirectly lessen ER stress in β cells and improve glycemic outcomes (Green & Olson, 2011). Table 2 summarizes molecular chaperones and antioxidants that alleviate ER stress and their effects on β cells.

#### Chemical chaperones and antioxidant strategies relieving *β*-cell ER and oxidative stress

TUDCA is a taurine conjugate of a hydrophobic bile acid that exhibits chemical chaperone and cytoprotective properties. In mouse studies, in non-obese diabetic models, intraperitoneal TUDCA at a total daily dose of 500 mg/kg during the prediabetic period alleviates β cell ER stress, rebalances the adaptive arm of the UPR, reduces islet inflammatory infiltration, lowers β cell apoptosis, and delays hyperglycemia (Engin et al., 2013). Earlier work showed that in obese and type 2 diabetic mice, intraperitoneal TUDCA reduces hepatic and systemic ER stress, improves insulin sensitivity, and enhances glycemic control, suggesting antidiabetic effects through strengthened ER folding capacity (Ozcan et al., 2006). In humans with type 2 diabetes or insulin resistance, oral TUDCA at a total of 1,750 mg per day for 4 weeks increases insulin sensitivity in skeletal muscle and liver. Although β cell ER stress markers were not directly measured, the systemic improvement aligns with the mouse mechanism (Kars et al., 2010). Mechanistically, TUDCA stabilizes the ER folding milieu and chaperone network, lowers PERK and IRE1 outputs, limits CHOP mediated apoptosis, and its antioxidant effects help interrupt the positive feedback between oxidative and ER stress, thereby directly protecting β cells and indirectly improving insulin action in extraislet tissues (Engin et al., 2013; Ozcan et al., 2006). In sum, effective mouse dosing commonly uses 500 mg/kg per day by intraperitoneal injection, while human studies have used 1,750 mg per day orally with metabolic benefit, with mouse data providing direct support for relief of β cell ER stress (Engin et al., 2013; Kars et al., 2010).

**Table 2:** Agents relieving ER stress and their functional impacts on β cells.

**Drug**	**Dose**	**Model**	**Effect**	**References**
TUDCA	250 mg/kg intraperitoneal, twice daily, total about 500 mg/kg per day, administered in the prediabetic phase	NOD mice prone to type 1 diabetes	Alleviated beta cell ER stress, reduced islet inflammation and apoptosis, delayed onset of hyperglycemia	Engin et al. (2013)
TUDCA	1,750 mg per day by mouth for 4 weeks	Adults with type 2 diabetes or insulin resistance in a randomized crossover study	Improved insulin sensitivity in skeletal muscle and liver as assessed by a hyperinsulinemic euglycemic clamp	Kars et al. (2010)
PBA	1 g per kg per day by mouth *via* gavage or drinking water for 20 to 28 days	Obese and type 2 diabetic mice including ob/ob and diet induced obesity models	Reduced hepatic and systemic ER stress and improved insulin sensitivity and glycemic control	Ozcan et al. (2006)
PBA	1 g per kg per day in drinking water for 12 weeks	Obese mice with human IAPP overexpression	Lowered fasting and postprandial glucose, reduced islet amyloid deposition, improved islet insulin secretion	De Pablo et al. (2021)
PBA	7.5 g per day by mouth for 2 weeks before lipid infusion	Healthy adults with insulin resistance induced by a 48-hour lipid infusion in a randomized crossover study	Partially reversed insulin resistance and improved the disposition index	Xiao, Giacca & Lewis (2011)
Melatonin	10 mg per kg per day by oral gavage for 15 days	Rats with type 2 diabetes	Decreased glucose and HbA1c, increased insulin, reduced pancreatic oxidative stress and apoptosis	Abdulwahab et al. (2021)
Quercetin	Endothelial cells received pretreatment with 25 µM quercetin	*In vitro* beta cell and endothelial cell coculture model of ER stress	Protected beta cells through nitric oxide to cGMP signaling and increased intracellular insulin and cGMP	Suganya et al. (2018)
Quercetin	Diet contained 1.5 g per kg of feed; the average intake was about 100 mg per kg body weight per day for 4 months	High fat diet plus low dose streptozotocin mouse model of type 2 diabetes	Improved glucose tolerance, reduced pancreatic iron deposition and lipid peroxidation, alleviated beta cell ferroptosis	Li et al. (2020)
Resveratrol	20 mg per kg per day by mouth for 12 weeks	db/db mice	Improved glucose tolerance, increased pancreatic weight and beta cell mass, reduced islet fibrosis and oxidative injury	Lee et al. (2012)

PBA is an orally available chemical chaperone that reduces intracellular unfolded protein burden and improves ER homeostasis. In a randomized cross over study in humans, healthy adults received 48 h of lipid infusion to induce insulin resistance and β cell dysfunction. Pretreatment with oral PBA at 7.5 g per day for 2 weeks partially reversed lipid induced insulin resistance and improved the disposition index that reflects coupling of secretion and sensitivity, indicating mitigation of lipotoxicity related β cell dysfunction and upward ER stress signaling (Xiao, Giacca & Lewis, 2011). In obese mice overexpressing human islet amyloid polypeptide and developing type 2 diabetes, oral PBA significantly lowered fasting and postprandial glycemia, reduced islet amyloid deposition, and improved islet insulin secretion, supporting *in vivo* relief of protein folding stress and secretory load (De Pablo et al., 2021). Broader metabolic study also shows that PBA and TUDCA reduce ER stress, restore whole body insulin sensitivity, and improve fatty liver in type 2 diabetic mice, revealing antidiabetic potential through enhanced ER adaptability (Ozcan et al., 2006). Taken together, Animal models typically employ oral dosing over several weeks with sustained glucose lowering and islet protection. The principal mechanism is reduction of unfolded protein accumulation, attenuation of excessive UPR activation and CHOP induction, and consequent direct relief of β cell ER stress while limiting lipotoxic injury.

Melatonin both scavenges free radicals and upregulates endogenous antioxidant systems, thereby lowering oxidative stress in islets and peripheral tissues. In a rat model of type 2 diabetes, oral melatonin at 10 mg/kg by daily gavage for 15 days significantly reduces blood glucose and glycated hemoglobin, increases insulin, decreases pancreatic oxidative stress and proinflammatory factors, and suppresses pancreatic cell apoptosis, indicating tissue level improvement in metabolism and survival (Abdulwahab et al., 2021). In high glucose induced β cell injury, studies comparing the impact of oxidative *versus* ER stress on glucose stimulated insulin secretion show that under chronic high glucose, ER stress contributes more critically to secretory defects, whereas melatonin primarily protects β cell viability by mitigating oxidative stress. These results suggest that combining melatonin with chaperone drugs that directly modulate the UPR may more effectively restore β cell function and secretion (Park et al., 2014). Additional *in vitro* data indicate that melatonin inhibits β cell apoptosis and early senescence, supporting the view that antioxidant and mitochondrial protection indirectly lessen the ER folding burden, reduce ER stress linked death signaling, and ultimately improve support for insulin biosynthesis and secretion (Park et al., 2014). Improvements in β cell morphology and function primarily depend on ER stress reduction secondary to antioxidant unloading and complement the direct UPR remodeling achieved by chemical chaperones.

Quercetin is a widely distributed dietary flavonoid that scavenges ROS and modulates ferroptosis. In a co culture system, endothelial cells pretreated with 25 micromolar quercetin protect β cells challenged by ER stress *via* NO signaling, increasing intracellular insulin and cyclic guanosine monophosphate levels. These findings indicate that quercetin can buffer ER stress at the microenvironmental level and improve secretory coupling (Suganya et al., 2018). Across β cell and islet models, quercetin confers dose dependent cytoprotection against H_2_O_2_ or high glucose injury, enhances mitochondrial biogenesis, and promotes insulin secretion, showing that it indirectly reduces ER stress by suppressing oxidative pressure and maintaining energetic homeostasis (Dhanya & Kartha, 2021). Recent work further shows that in type 2 diabetes settings, quercetin alleviates β cell ferroptosis, decreases pancreatic iron deposition and lipid peroxidation, and thus inhibits upstream triggers of ER folding disturbances, preserving β cell survival and function (Li et al., 2020). In summary, cell studies commonly use micromolar concentrations. The key mechanisms are reduction of ROS and iron dependent lipid peroxidation, support of mitochondrial function and secretion coupling, and attenuation of ER stress outputs through local microenvironmental signaling. Quercetin therefore operates as a composite intervention that emphasizes antioxidant and metabolic stabilization with secondary relief of ER stress.

Resveratrol is a polyphenolic natural compound with pronounced antioxidant and antiinflammatory activity that activates sirtuin 1 and AMP activated protein kinase. In Lepr db/db mice, oral resveratrol at 20 mg/kg daily for 12 weeks improves glucose tolerance, increases pancreatic weight and β cell mass, and reduces islet fibrosis and oxidative injury, demonstrating clear *in vivo* protection of islet structure and function (Lee et al., 2012). In another combined animal and cell study, resveratrol lowered fasting glucose and lipid peroxidation in Lepr db/db mice and protected β cells from oxidative stress *in vitro*, suggesting that decreased ROS generation and strengthened antioxidant defenses indirectly reduce the ER stress amplification loop (Minakawa et al., 2011). Consistent with these findings, in streptozotocin–nicotinamide–induced diabetic rats, low dose oral resveratrol (5 mg/kg for 30 days) lowers circulating and pancreatic levels of TNF-α, IL-1β, IL-6, NF-κB p65 and nitric oxide, attenuates hyperglycemia mediated oxidative stress, restores enzymatic and non-enzymatic antioxidant defenses, and preserves β cell ultrastructure, directly linking cytokine suppression and oxidative stress relief to maintenance of β cell function (Palsamy & Subramanian, 2010). In a type 1 diabetes model induced by multiple low dose streptozotocin injections in Balb/c mice, resveratrol at 50 mg/kg ameliorates hyperglycemia, improves serum insulin and β cell mass, reduces oxidative imbalance, and reverses CXCL16, oxidized LDL, tissue factor and autophagy marker alterations in pancreatic tissue, indicating that resveratrol also counteracts chemokine and ox LDL driven autophagy mediated β cell death (Darwish et al., 2021). Considering the coupling between oxidative and ER stress in β cells, reducing oxidative load diminishes folding errors and excessive UPR activation, suppresses CHOP linked death pathways, and preserves the architecture and efficiency of the secretory apparatus. This inference aligns with mechanistic studies of stress coupling in β cells (Oslowski & Urano, 2011). It should be noted that although resveratrol shows consistent metabolic and morphologic benefits in animals, direct clinical evidence for lowering β cell ER stress markers remain limited. Its benefits likely reflect indirect ER relief *via* antioxidant unloading and metabolic reprogramming, and combination with chemical chaperones warrants evaluation.

These lines of evidence indicate that chemical chaperones such as TUDCA and PBA directly relieve β cell ER stress and deliver robust metabolic benefits, whereas antioxidants and multitarget natural compounds represented by quercetin and resveratrol more often reduce oxidative load, improve mitochondrial and iron metabolism, and optimize the cellular microenvironment to indirectly lessen ER stress. Choice of specific agents and doses should integrate evidence level and population characteristics, and effective mouse doses should not be extrapolated directly to humans. Based on the mechanistic chain and multi-source evidence, building a combined intervention framework with direct ER stress modulation at its core and antioxidant or metabolic reprogramming as adjuncts may more effectively protect β cell survival and function and slow diabetes progression.

#### Gene editing strategies targeting β-cell stress pathways

Cai et al. (2020) performed a genome-wide CRISPR loss-of-function screen under conditions of strong autoimmune attack and observed a marked enrichment of Rnls-deficient cell clones, suggesting that RNLS is one of the key nodes limiting β-cell survival and thus represents a potential target for cytoprotective intervention. In NIT-1 β cells, Rnls knockout enhances cellular tolerance to thapsigargin- and tunicamycin-induced endoplasmic reticulum ER stress and attenuates cell injury caused by combined IL-1β and IFN-γ stimulation (Cai et al., 2020). Mechanistically, Rnls loss is associated with an elevated threshold for UPR activation, reflected by reduced activation of the PERK–eIF2α, IRE1α and ATF6 branches, accompanied by downregulation of CHOP and Txnip. In parallel, NRF2 expression is upregulated and β cells display increased resistance to oxidative stress (Cai et al., 2020). *In vivo*, insulin promoter–driven *in situ* editing of Rnls maintains higher insulin expression in transplanted islets exposed to a strong autoimmune milieu, and targeting Rnls per se does not impair the insulin secretory capacity of islet cells (Cai et al., 2020). In human iPSC-derived β-like cells, RNLS knockout similarly does not alter the expression of differentiation markers or glucose-stimulated insulin secretion GSIS, but reduces thapsigargin-induced ER-stress–related cell death (Cai et al., 2020). At the pharmacological level, structural modelling and *in vitro* binding assays indicate that pargyline can interact with RNLS. In diabetic NOD mouse β-cell transplantation models, oral pargyline improves graft survival and reverses hyperglycemia, and in both cyclophosphamide-accelerated NOD and anti–PD-1-induced autoimmune diabetes models it prevents or delays disease onset, while also reducing thapsigargin-induced cell death in stem cell–derived β-like cells, indicating that pargyline partially phenocopies the protective effects of RNLS deficiency at the functional level (Cai et al., 2020).

In line with the RNLS axis, Bompada et al. (2016) used CRISPR/Cas9 to delete EP300 in INS1 832/13 cells and found that under high-glucose conditions H3K9ac and H4ac at the Txnip promoter and first coding region are markedly reduced, accompanied by decreased Txnip mRNA and protein expression. In EP300-deficient cells, glucose-induced apoptosis is substantially reduced and glucose-stimulated insulin secretion is enhanced, effects that the authors primarily attribute to Txnip downregulation (Bompada et al., 2016). Previous studies further show that IRE1α signalling can induce Txnip and contribute to ER-stress–related cell death (Lerner et al., 2012). At the same time, TXNIP functions as an inhibitory binding partner of thioredoxin and thereby perturbs cellular redox homeostasis (Nishiyama et al., 1999; Patwari et al., 2006). In β-cell models, increased Txnip levels are associated with elevated ROS and loss of GSIS, whereas Txnip downregulation lowers ROS and improves GSIS (Rani et al., 2010). In addition, TXNIP has been reported to drive β-cell apoptosis predominantly through the intrinsic mitochondrial pathway (Chen et al., 2008). In a human iPSC-based model of INS mutation–related neonatal diabetes, investigators established isogenic control lines by CRISPR-Cas9 correction of the INS mutation and showed that INS-mutant β-like cells exhibit increased ER stress and reduced proliferation, suggesting that such mutations more likely promote β-cell failure by limiting expansion of β-cell mass rather than by markedly increasing apoptosis (Balboa et al., 2018).

Taken together, these data indicate that CRISPR-mediated gene editing and epigenetic modulation can be used to intervene at multiple levels in β-cell stress susceptibility. Targeting RNLS, TXNIP and their upstream epigenetic regulators has the potential to raise the tolerance threshold to ER and oxidative stress without overtly disturbing β-cell identity or secretory function, thereby reshaping β-cell survival and function under glucolipotoxic and inflammatory conditions and providing combinable intervention strategies for islet replacement therapy and β-cell protection.

### Conclusion and future perspective

This review centers on the redox imbalance and ER stress faced by pancreatic β cells under high secretory demand, and highlights how these two pressures amplify one another to shape islet homeostasis. Because β cells have limited antioxidant redundancy, disruption of calcium and redox coupling between mitochondria and the ER lowers the coupling of proinsulin folding with secretion, which manifests as weakened glucose stimulated secretion and a regressive functional phenotype. As stress persists, the adaptive UPR shifts from protection to injurious output, which induces dedifferentiation and activates apoptotic pathways, leading to a decline in β cell number and in islet functional reserve. Increases in lipid and nucleic acid oxidation often precede structural damage and run in parallel with diminished secretion, and can be used as early risk warnings and quantitative indicators of disease course. At the therapeutic level, antioxidants clear ROS and bolster endogenous defenses, lower peroxidative burden, and disrupt the positive feedback loop between redox pressure and ER stress. In this way they relieve folding defects and functional injury and restrain cell death. When used together with chemical chaperones, they may further expand folding and clearance capacity, stabilize key folding enzymes, and ultimately preserve β cell function and number in complex stress settings while slowing diabetes progression.

Future work should first test causality at key nodes in human islets and organoids, and incorporate early readouts such as the proinsulin to insulin ratio, 4-HNE protein adducts, 3-nitrotyrosine, and 8-oxoguanine into indicator panels. Second, studies should define with precision the thresholds and time windows at which each UPR branch switches from adaptation to injury, including PERK, eIF2α, ATF4, CHOP, as well as IRE1α, XBP1, and ATF6. The goal is to preserve protective outputs that expand folding and clearance while suppressing proapoptotic signaling. Third, along the axis of coupling between the ER and mitochondria, work should resolve how calcium dynamics and redox flux constrain secretory coupling, and clarify the relative contributions of NOX2 and mitochondrial ROS in physiology and disease. At the therapeutic level, systematic comparisons of monotherapy and combinations built around TUDCA, PBA, and antioxidants such as melatonin, quercetin, and resveratrol should quantify additive effects on lowering CHOP and restoring the XBP1s, and should define order, dose, and safety windows. Studies should also test the value of pharmacologic activation of Nrf2 and the peroxiredoxin and thioredoxin axis, together with inhibition of TXNIP. Finally, in models that combine hyperglycemia, hyperlipidemia, and inflammation, single cell multiomics integrated with functional readouts should be used to track transitions among adaptation, dedifferentiation, and apoptosis, in order to generate biomarkers and endpoints ready for small clinical studies.

## Additional Information and Declarations

### Data Availability

The following information was supplied regarding data availability: This is a literature review.
